# Global Outbreak Alert and Response Network (GOARN) focal point engagement meeting with partners in Japan

**DOI:** 10.5365/wpsar.2024.15.5.1100

**Published:** 2024-01-30

**Authors:** Haruka Iwasaki, Sharon Salmon, Yukimasa Matsuzawa, Sangnim Lee, Kanae Takagi, Hidetoshi Nomoto, Masahiro Ishikane, Mugen Ujiie, Norio Ohmagari

**Affiliations:** a Disease Control and Prevention Center, National Center for Global Health and Medicine, Tokyo, Japan.; b World Health Organization Regional Office for the Western Pacific, Manila, Philippines.; c Indo-Pacific Centre for Health Security, Department of Foreign Affairs and Trade Australia, Canberra, Australia.; d UNSW Medicine, School of Public Health and Community Medicine, University of New South Wales, Sydney, Australia.; e Global Outbreak Alert and Response Network, World Health Organization, Geneva, Switzerland.; f Global Outbreak Intelligence, Capacity Building and Deployment Coordination Center, Disease Control and Prevention Center, National Center for.; Global Health and Medicine, Tokyo, Japan.; g Department of Epidemiology and Clinical Research, The Research Institute of Tuberculosis, Japan Anti-Tuberculosis Association, Tokyo, Japan.; h Department of Respiratory Medicine, National Center for Global Health and Medicine, Tokyo, Japan.; i WHO Collaborating Centre for Prevention, Preparedness and Response to Emerging Infectious Diseases, Disease Control and Prevention Center, National Center for Global Health and Medicine, Tokyo, Japan.

In response to the evolving 2015 Ebola virus disease outbreak in West Africa, the Government of Japan formulated an Action Plan for Strengthening Measures on Emerging Infectious Diseases (*1*) to promote international cooperation, testing and research systems in Japan and strengthen contributions and human resource capacity for supporting domestic and international public health emergencies.

To update and further this work, the first Global Outbreak Alert and Response Network (GOARN) focal point engagement meeting in Japan was held on 18 November 2022. The meeting was hosted by the National Center for Global Health and Medicine (NCGM) and supported by the Ministry of Health, Labour and Welfare, Japan, the World Health Organization (WHO) headquarters and the WHO Regional Office for the Western Pacific. The programme for the meeting aimed to orient participants to GOARN areas of work, determine priority actions to implement the new GOARN Strategy 2022–2026 (*2*) and share partner experiences to identify strengths and limitations in network engagement including deployment. The purpose of the meeting was to gather and connect GOARN partner focal points from Japan to strengthen collaboration between partners, improve network participation, implement activities and increase the number of ready-to-deploy national experts to support international outbreak response.

The half-day programme consisted of four sessions that were delivered with simultaneous translation in Japanese and English. The meeting was attended by 38 participants, including 15 GOARN focal points or designees representing 15 of the 17 partners located in Japan, who attended both virtually and physically in Shinjuku Ward, Tokyo (**Table 1**). Speakers, presenters, panellists and facilitators comprised GOARN partners, the GOARN Steering Committee and WHO.

**Table 1:** Characteristics of participating GOARN partner institutions and individual participants

Participating GOARN partner institutions		No.
**GOARN partner institutions in Japan**	**Physical/on-site** **Online**	**12** **3**
-	**Total**	**15**
GOARN partner institutions’ focal point attendees	Physical/on-site Online	8 2
GOARN partner institutions’ focal point designated attendees	Physical/on-site Online	4 1
-	**Total**	**15**
**Participants**		**No.**
Participant attendance by modality	Physical/on-site Online	25 13
-	**Total**	**38**
Participants’ institutional affiliation	Medical institute/health-care facility Government organizations Academic/research or education International or official development assistance implementing agencies Nongovernmental organization	15 10 9 3 1
-	**Total**	**38**

GOARN: Global Outbreak Alert and Response Network.

The first session oriented participants to GOARN and its strategy for 2022–2026, (*2*) including GOARN activities within the WHO Western Pacific Region, deployment mechanisms and training programmes.

The second session covered the role of the GOARN focal points, outlining expectations and tips on improving partner engagement. A moderated panel discussion enabled the exchange of experiences on effectively sharing GOARN communications within institutions, including ways to gain institutional support for participation during international outbreak response. Panellists shared methodologies for identifying individuals suitable for deployment and for strengthening response capacity by building a cadre of ready-to-respond young professionals through mentorship.

During session three, speakers from partners in Japan shared experiences with delivering the GOARN trainings at local, national and international levels and presented plans for more trainings. Participants heard individual accounts of how deployment through GOARN’s international outbreak response enhanced public health career prospects.

In the final session, participants worked in small groups to develop an action plan for activities with partners in Japan. They were reminded of the obstacles to deployment (*3*) and discussed the optimal institutional capacity for active involvement in GOARN, and how to improve international deployment capacity in response to public health emergencies.

The partners confirmed GOARN’s important role in enhancing global health security through emergency response coordination. GOARN helps to build capacity and respond swiftly to outbreaks by providing real-time information sharing, technical assistance and technical expert deployment to the field.

Participants deemed GOARN focal points crucial as primary contact persons within institutions, facilitating communications and information, and coordinating support to disease outbreaks and health emergencies. Focal points requested more engagement and collaboration across larger institutions with more GOARN involvement. Ideas for opportunities to collaborate included co-hosting or participating in trainings and joining deployment debriefings and information sessions on Japan’s GOARN roster system.

An online post-meeting evaluation was conducted with a response rate of 47% (18/38). Participants reported a better understanding of GOARN’s activities and processes and were highly satisfied with the meeting’s programme and delivery. Participants agreed that GOARN was crucial for preparedness for and response during global infectious disease outbreaks. The three most valued activities in the programme were networking with other partners, understanding the GOARN deployment mechanism and listening to deployment experiences. Participants requested more opportunities to network with partners from Japan and the Western Pacific Region and share deployment experiences to better understand the professional and field skills required to assist in international response.

A key outcome of the meeting was partners’ agreement to participate in an annual GOARN focal point engagement meeting to review Network participation, enhance Japan’s GOARN deployment roster and collaborate within the Network to strengthen health emergency preparedness and response domestically and internationally.
